# Dexmedetomidine Protects Against Oxygen–Glucose Deprivation-Induced Injury Through Inducing Astrocytes Autophagy via TSC2/mTOR Pathway

**DOI:** 10.1007/s12017-019-08576-0

**Published:** 2019-10-25

**Authors:** Chen Zhu, Quan Zhou, Cong Luo, Ying Chen

**Affiliations:** 1 Department of Anesthesiology, Nanfang Hospital, Southern Medical University, 1838 Guangzhou Avenue N, Guangzhou, 510515 Guangdong China; 2 Department of Anesthesiology, The Sixth People’s Hospital of Chengdu, 16 Jianshe Street S, Chengdu, 610051 Sichuan China

**Keywords:** Dexmedetomidine, Ischemic stroke, Neuroprotection, Astrocytes, Autophagy

## Abstract

Although there is an increment in stroke burden in the world, stroke therapeutic strategies are still extremely limited to a minority of patients. We previously demonstrated that dexmedetomidine (DEX) protects against focal cerebral ischemia via inhibiting neurons autophagy. Nevertheless, the role of DEX in regulating astrocytes autophagic status in oxygen–glucose deprivation, a condition that mimics cerebral ischemia, is still unknown. In this study, we have shown that DEX and DEX + RAPA (autophagy inducer) increased viability and reduced apoptosis of primary astrocytes in oxygen–glucose deprivation (OGD) model compared with DEX + 3-methyladenine (3-MA) (autophagy inhibitor). DEX induced the expression of microtubule-associated protein 1 light chain 3 (LC3) and Beclin 1, while reduced the expression of p62 in primary cultured astrocytes through induction of autophagy. In addition, DEX enhanced the expression of tuberous sclerosis complex 2 (TSC2) in primary cultured astrocytes, while reduced the expression of mammalian target of rapamycin (mTOR). In conclusion, our study suggests that DEX exerts a neuroprotection against OGD-induced astrocytes injury via activation of astrocytes autophagy by regulating the TSC2/mTOR signaling pathway, which provides a new insight into the mechanisms of DEX treatment for acute ischemic injury.

## Introduction

Autophagy is a major cellular pathway for the degradation of long-lived proteins and cytoplasmic organelles through an autophagosome-lysosome pathway to help cells adapt to changing nutrient conditions and maintain their homeostasis (Levine and Klionsky 2004; Pla et al. 2016). After cerebral ischemia, autophagy of neurons, astrocytes and microglia are both activated (Tian et al. 2010). Adopting a permanent MCAO mice model, Qin et al. also found that induced astrocytes autophagy may contribute to cerebral ischemic injury (Qin et al. 2010). However, other research found that 3-methyladenine (3-MA), autophagy inhibitor, could increase cell death in hypoxic astrocytes accompanied with accumulating autophagic proteins LC3 II as well as autophagic substrate p62 (Bao et al. 2017). One reason for this discrepancy is the fact that the ischemic reperfusion injury is time-dependent. A recent study found that 3-MA increased astrocytes apoptosis following 1 or 4 h OGD. However, once the duration of OGD prolonged to 8 or 24 h, 3-MA could inhibit extrinsic and intrinsic apoptotic pathway (Gabryel et al. 2017; Perez-Alvarez et al. 2018). More importantly, autophagy also plays a complex and bi-directional effect after cerebral ischemia, which may rely on the extent of brain injury, the autophagic burden, the timing of intervention of s autophagic status, etc. Thus, the role of astrocytes autophagy is still in need of more research to clarify.

Dexmedetomidine (DEX), a selective α_2_-adrenoceptor agonist, is a useful sedative in the intensive care unit and anesthesia. The sedation of DEX is easy to arouse and DEX is associated with a lower rate of postoperative delirium than other sedatives (Mu et al. 2015). Apart from above, there is increasing evidences indicating that α_2_-agonists weaken the morphologic and functional destructive effect after ischemic reperfusion injury, including cerebral ischemia, cardiac arrest, and kidney ischemic injury (Gu et al. 2011; Luo et al. 2017; Sun et al. 2017). Previous reports demonstrated that DEX protects against cerebral ischemic reperfusion injury in vitro and in vivo, and this effect is mediated via increases in the expression of pERK1 and 2 independents of α_2_ AR activation (Ma et al. 2004; Dahmani et al. 2008). DEX also protects against OGD-induced injury in rat C6 glioma cells via PI3K/AKT pathway (Zhang et al. 2012). In addition, DEX-induced activated astrocytes may promote the release of glial cell line-derived neurotrophic factor (GDNF) to rescue neurons after ischemia injury (Yan et al. 2011; Degos et al. 2013). Previous research suggested that DEX showed a protective effect on lung and kidney tissue throughout autophagy cascade in ischemia reperfusion (Lempiainen et al. 2014; Xie et al. 2015). In our previous research, we found that DEX could inhibit neuronal autophagy through HIF-1α to produce neuroprotection after cerebral ischemia (Luo et al. 2017). Nevertheless, the effect of DEX on astrocyte’s autophagy in conditions similar to cerebral ischemia is still unknown.

In present study, we hypothesized that DEX may regulate the astrocytes’ autophagy and then could increase the survival or decrease the apoptosis of astrocytes exposed to OGD. To test our hypothesis, we first examined the effect of DEX on the survival and apoptosis of astrocytes following OGD. Then we investigated the autophagy level of astrocytes exposed to OGD when treated by DEX, or combined with autophagic inhibitor, 3-MA or inducer, RAPA. Our results showed that DEX could increase and decrease the viability and apoptosis of astrocytes exposed to OGD ,respectively, which effect could be inhibited by 3-MA while enhanced by RAPA. Furthermore, we found DEX-induced autophagy in astrocytes may occur through TSC2/mTOR pathway.

## Materials and Methods

All animals used in present study were provided by the Southern Medical University Administrative Panel on Laboratory Animal Care and all animal experiments were carried out according to the guidelines of Animal Use and Care of Southern Medical University.

### Primary Astrocyte Culture

Post-natal within 2 days C57/BL6 mice were purchased from the Experimental Animals Center, Southern Medical University. Cerebral cortices were collected, minced, and then digested with 0.25% trypsin (Gibco, France) and DNase (Sigma-Aldrich, USA) at 37 °C for 5 min. Dissociated cerebral cortical cells were mixed with DMEM-F12 containing 10% fetal bovine serum and a density of 5 × 10^5^ cells/cm_2_ were seeded in each well of standard 25 cm^2^ flasks. The cells were cultured at 37 °C with a humidified 5% CO_2_. Culture media was replaced 1 day later, and then twice a week. After 10 days of incubation, microglia and oligodendrocytes were removed by mild shaking for 15–18 h at 200 rpm. The remaining astrocytes were detached with 0.25% trypsin and reseeded onto the flasks again. GFAP staining purity of more than 95% of the astrocytes cells were collected for the following experimental research (Yan et al. 2011).

### Determination of the Optimal Oxygen–Glucose Deprivation (OGD) Duration, DEX Concentration, and Re-oxygenate Duration

To mimic the oxygen–glucose deprivation environment, the astrocytes culture medium was removed and replaced with glucose-free Earle’s balanced salt solution (EBSS) and then incubated in a hypoxic chamber (Forma Scientific, USA) which was continuously supplied with a gas mixture of 95% N_2_ and 5% CO_2_ at 37 °C (Yan et al. 2011). EBSS group was exposed to EBSS in the standard incubator. OGD was terminated by replacing the medium to standard astrocytes culture medium in the normoxic incubator. Dexmedetomidine (DEX), a molar weight of 236.7 g/mol for dexmedetomidine-HCl (100 μg/ml, Hengrui, Jiangsu, China), was added to this experimental medium after ischemia. To determine the optimal OGD duration and DEX concentration, different OGD duration (1, 2, 3, and 4 h) and different final concentrations of DEX (0.5 μM, 1.0 μM, 2.0 μM, and 5.0 μM) were administrated to determine the optimal concentration. Once the optimal OGD duration and DEX concentration are determined, the viability of astrocyte experienced OGD at different reoxygenation timepoint (1, 3, 6, 12, and 24 h) were observed to determine the best reoxygenation time. At the end of OGD, the treatment groups were randomly divided into the following groups: control group, OGD group, OGD + DEX group, OGD + 3-MA group, OGD + DEX + 3-MA group, OGD + RAPA group, and OGD + DEX + RAPA group. The final concentration of 3-MA (10 mM) (Qin et al. 2010) or RAPA (1 μM) (Janen et al. 2010) effectively inhibited or enhanced the activation of autophagy as evidenced by LC3 expression, respectively. All experiments were at least duplicated three times biologically.

### Assessment of Astrocytes Viability and Apoptosis After OGD

CCK-8 assay (CCK-8; Dojin Laboratories, Japan) was adopted to evaluate the viability of different groups. Briefly, astrocytes were cultured on 96-well plates and then treated with 10 μl CCK-8 reagent for 2 h at 37 °C with a humidified 5% CO_2_. Results were visualized at 450 nm by the microplate reader (BioTek, USA). To assess the apoptotic status, the culture medium of the astrocytes was removed, then the astrocytes were washed twice with EBSS and stained with 5 ml of Annexin-V-Fluorescein isothiocyanate (FITC) and 10 ml of propidium iodide (PI). After 10 min incubation in the dark at room temperature, cells were analyzed via flow cytometry (BD bioscience, USA).

### Immunofluorescence

After removing the culture medium, the astrocytes were fixed in 4% methanol for 15 min and permeabilized with 0.25% Triton for 10 min. Then cells were blocked with 2% Bovine Serum Albumin (BSA, Sigma) for 1 h. And the cells were treated with primary antibodies: Beclin1 (Abcam, AB_1850830) and GFAP (Invitrogen, AB_10013482) antibodies were incubated at 4 °C overnight. After washing three times with PBST (phosphate-buffered saline with 0.1% Tween), the cells were incubated with a mixture of anti-goat(Invitrogen, AB_631727) and anti-rabbit antibodies (Invitrogen, AB_2651036) for 1 h. 4,6-diamidino-2-phenylindole (DAPI, USA) was used to stain the cells nuclei for 5 min in the dark. Finally, the stained cells were visualized using a fluorescent microscopy (Nikon ECLIPSE 80i, Japan).

### Immunoblotting

Cell samples were directly homogenized in lysis buffer, crushed by ultrasound with a gentle power and then reacted on ices for 20 min, followed by centrifuged and collected supernatant at − 80 °C. We determined the protein concentrations with a BCA protein assay kit (Pierce, 23225) before immunoblotting. Separation of proteins was done with differences in their molecular weights by electrophoresis, then transferred to PVDF membranes. After blocking with 5% fat free milk, the membranes were incubated with a TSC2 (Cell Signaling, AB_564043), and p62 (Cell Signaling, AB_2085095), and LC3 (Santa Cruz Biotechnology, AB_1962479), and Beclin 1 (Abcam, AB_1641491), and 4EBP1, p-4EBP1 (SIGMA, AB_2097980, AB_10557439), and mTOR, p-mTOR (SIGMA, AB_2231882, AB_2231882), and S6K1, p-S6K1 (SIGMA, AB_2569704, AB_1620652), and β-actin (Santa Cruz Biotechnology, AB_626632). Blots were visualized using an Odyssey infrared imaging system (LI-COR Biosciences, USA) and analyzed using Image J.

### Statistical Analysis

Data are shown as mean ± SEM, *n* = 3. Three or more groups comparisons were analyzed by one-way analysis of variance (ANOVA) followed by Tukey post hoc test. And we used the unpaired *t* test for only two groups comparisons. The significant statistical differences were defined as *P* < 0.05. All data were analyzed using SPSS 20.0 Statistics (IBM Corp).

## Results

### DEX Increased Viability of Astrocytes Following OGD

**Fig. 1:**
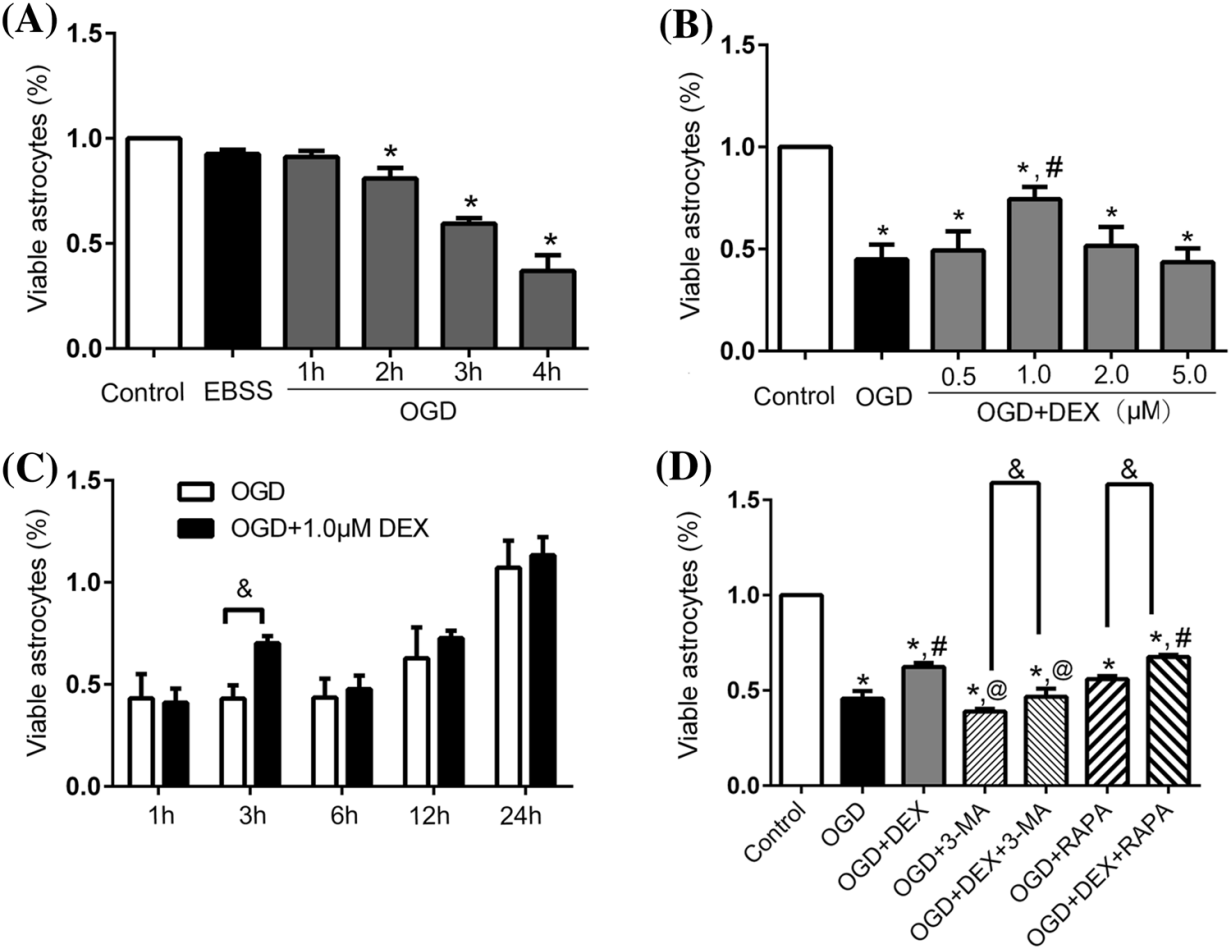
Effect of different OGD duration, dexmedetomidine concentration, and treating time on primary cultured astrocytes viability. **a** Astrocytes were subjected to OGD injury at 1, 2, 3, 4 h. **b** Dexmedetomidine (0.5, 1.0, 2.0 and 5.0 μM) was administrated after 3 h OGD. **c** Dexmedetomidine (1 μM) treated astrocytes 1, 3, 6, 12, and 24 h after 3 h OGD. **d** Viability of astrocyte treated with dexmedetomidine, 3-MA, RAPA and their different combinations after 3 h OGD. *Compared with Control group, *P *< 0.05; ^#^Compared with OGD group, *P *< 0.05; ^@^Compared with OGD + DEX group, *P *< 0.05; ^&^Compared between the two indicated groups, *P *< 0.05

To clarify the protective effect of DEX, 4 h OGD was chosen for all the following experiments (Fig. 1a). Then, in the following experiments, our results suggested that 1 μM DEX showed more protection than 0.5 μM. However, DEX did not show a dose-dependent protective effect because 2 and 4 μM DEX did not show any significant protective effect (Fig. 1b). Furthermore, the optimal reoxygenation duration were screened and only 3 h reoxygenation showed a significant increase viability when compared with the corresponding OGD group (Fig. 1c).

### DEX may Inducing Autophagy to Increase Astrocytes’ Viability

To investigate the potential mechanism of DEX protection on astrocytes exposure to OGD, we further investigated whether autophagy plays a role in the DEX protection of astrocytes after OGD. Our results indicated that DEX significantly increased the viability of astrocytes after OGD, whereas inhibiting autophagy using 3-MA decreased the viability of astrocytes after OGD and DEX could partly reverse the effect of 3-MA. Moreover, inducing autophagy using RAPA increased the viability of astrocytes after OGD and DEX could further increase the viability (Fig. 1d). These data indicated that DEX may induce autophagy to increase the viability of astrocytes after OGD.

### DEX Inhibited Apoptosis of Astrocytes Following OGD

**Fig. 2:**
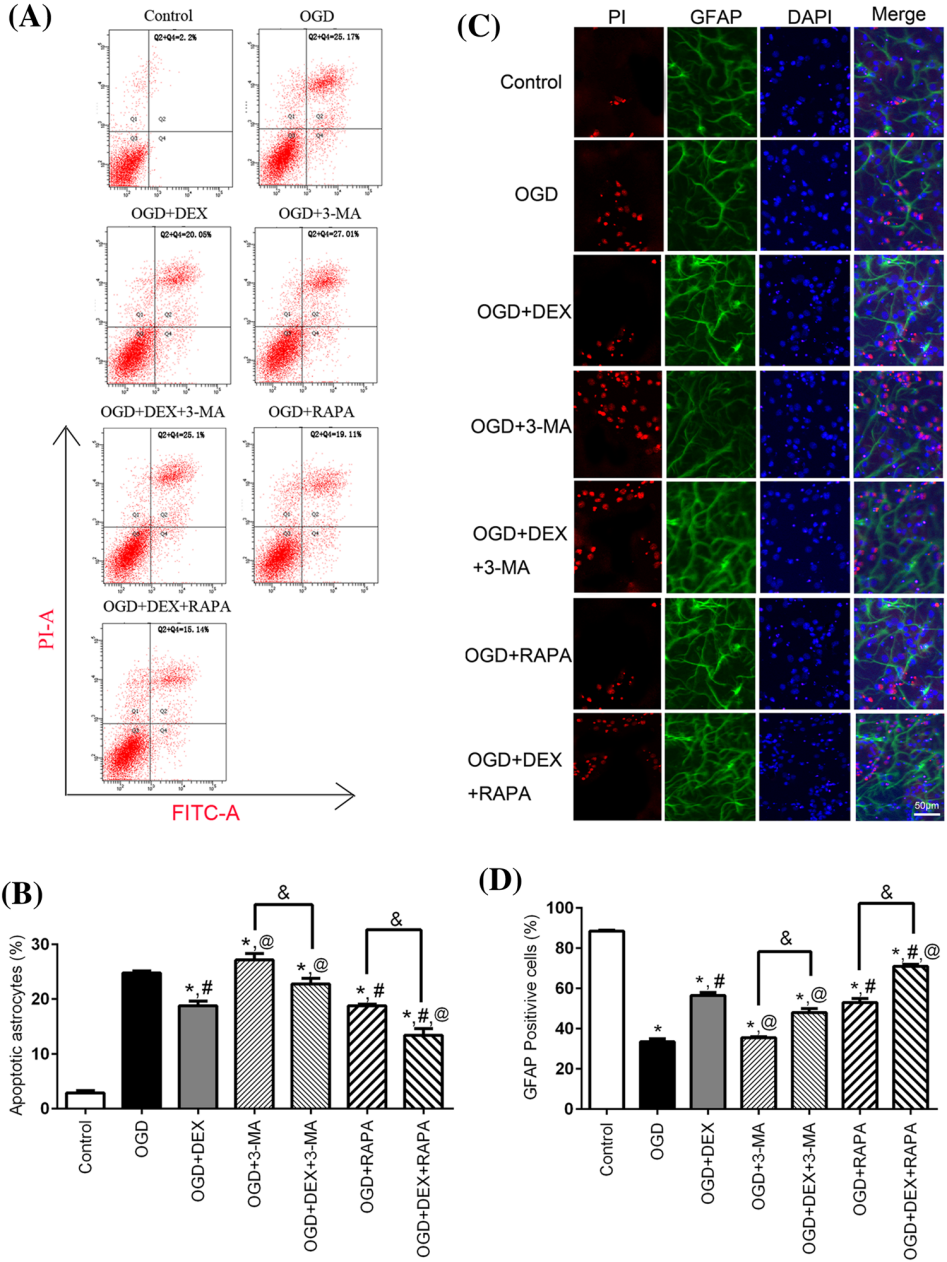
Effect of dexmedetomidine, 3-MA, and rapamycin treatment on primary cultured astrocytes apoptosis and viability after OGD. **a** Representative flow cytometry images of each group. **b** Percentage of apoptotic astrocytes following OGD. **c** Astrocyte immunofluorescent staining (DAPI, blue; GFAP, green, PI). **d** Percentage of GFAP and DAPI double positive cell following OGD. *Compared with Control group, *P *< 0.05; ^#^Compared with OGD group, *P *< 0.05; ^@^Compared with OGD + DEX group, *P *< 0.05; ^&^Compared between the two indicated groups, *P *< 0.05

We further investigated the effect of DEX on apoptosis of astrocytes exposed to OGD. Representative flow cytometric images from each group are shown (Fig. 2a). Our results found that DEX, RAPA, and DEX + RAPA significantly inhibited apoptosis of astrocytes following OGD compared with OGD. However, 3-MA increased the astrocytes apoptosis cells following OGD (Fig. 2b). Furthermore, we also performed immunofluorescence staining and counted GFAP/PI double positive cell of each group to confirm the effect of DEX on apoptosis of astrocytes exposed to OGD. Our cytometry and immunofluorescence results both found that DEX, RAPA, and DEX + RAPA decreased astrocytes apoptosis exposed to OGD, and 3-MA could partially reverse the effect (Fig. 2c, d).

### DEX Augmented Autophagy of Astrocytes Following Exposure to OGD

**Fig. 3:**
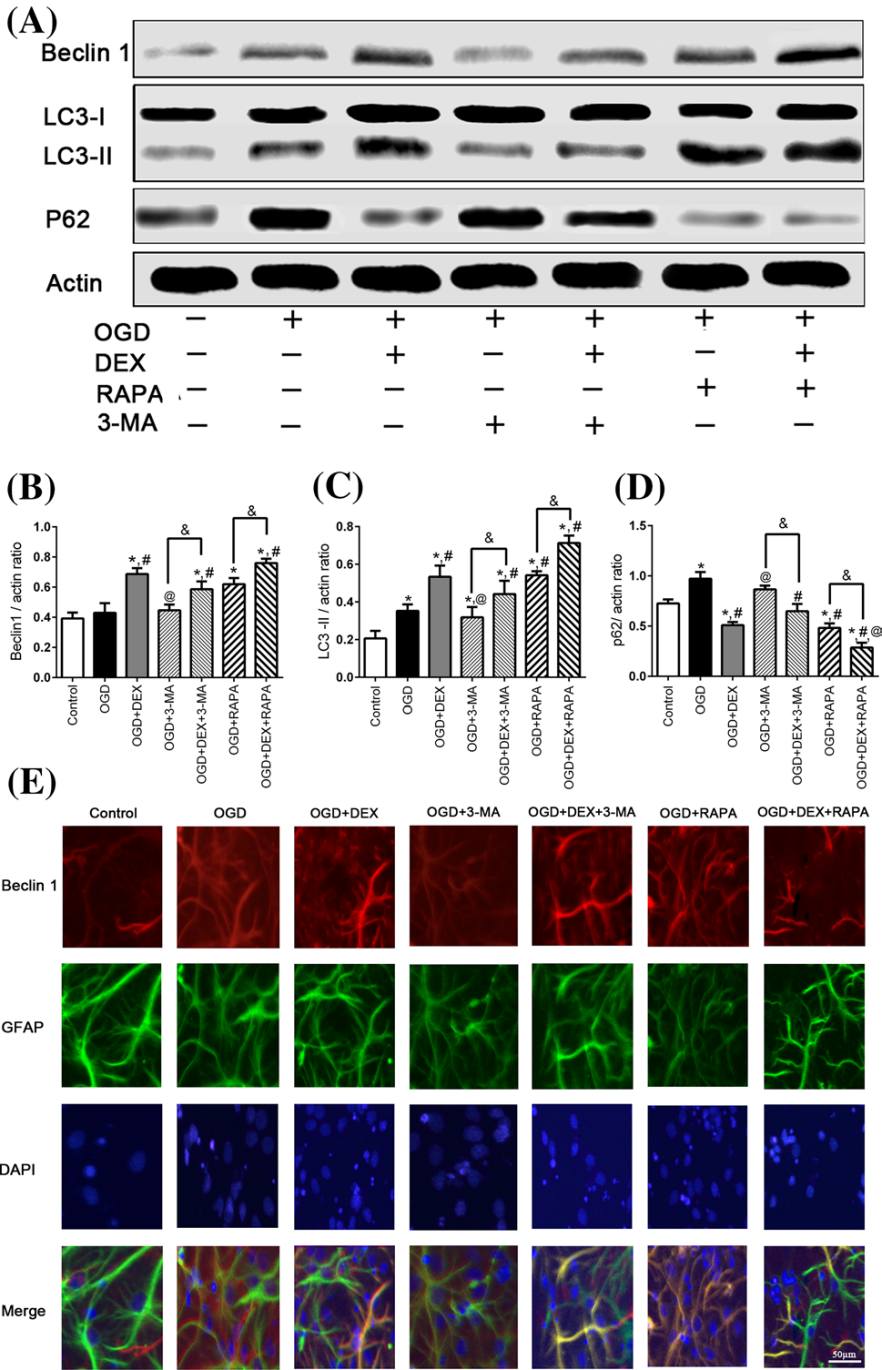
Dexmedetomidine treatment increased Beclin 1 and LC3 expression, while decreased the p62 expression. **a** Representative immunoblotting image of Beclin 1, LC3 I/II, and P62 expression when treated with dexmedetomidine (DEX) alone or in combination with 3-MA, rapamycin (RAPA) following OGD. b–d Dexmedetomidine treatment increased Beclin 1 and LC3 expression, while decreased p62 expression following OGD. **e** Representative immunofluorescent image of Beclin 1 of each group. *Compared with Control group, *P *< 0.05; ^#^Compared with OGD group, *P *< 0.05; ^@^Compared with OGD + DEX group, *P *< 0.05; ^&^Compared between the two indicated groups, *P *< 0.05

We further confirmed the expression of autophagy-related proteins including p62, LC3, Beclin1 in primary astrocytes from different groups via immunoblotting (Fig. 3a). Our results showed that DEX and RAPA could significantly increase LC3-II and Beclin 1 expression, while decrease the P62 expression. And when combined with RAPA, these autophagy-related proteins showed a more obvious inclination. However, 3-MA could produce the opposite trend of protein expression, DEX partly reverses 3-MA effect (Fig. 3b–d). Our further results of immunofluorescence also found that the fluorescent intensity of Beclin 1 was elevated when treated by DEX, RAPA, and DEX combined with RAPA (Fig. 3e). And the reversal of the decreasing effect of 3-MA on Beclin 1 by DEX was more obvious (Fig. 3e).

### DEX Regulated Autophagy via TSC2/mTOR Pathway in Astrocytes Following OGD

**Fig. 4:**
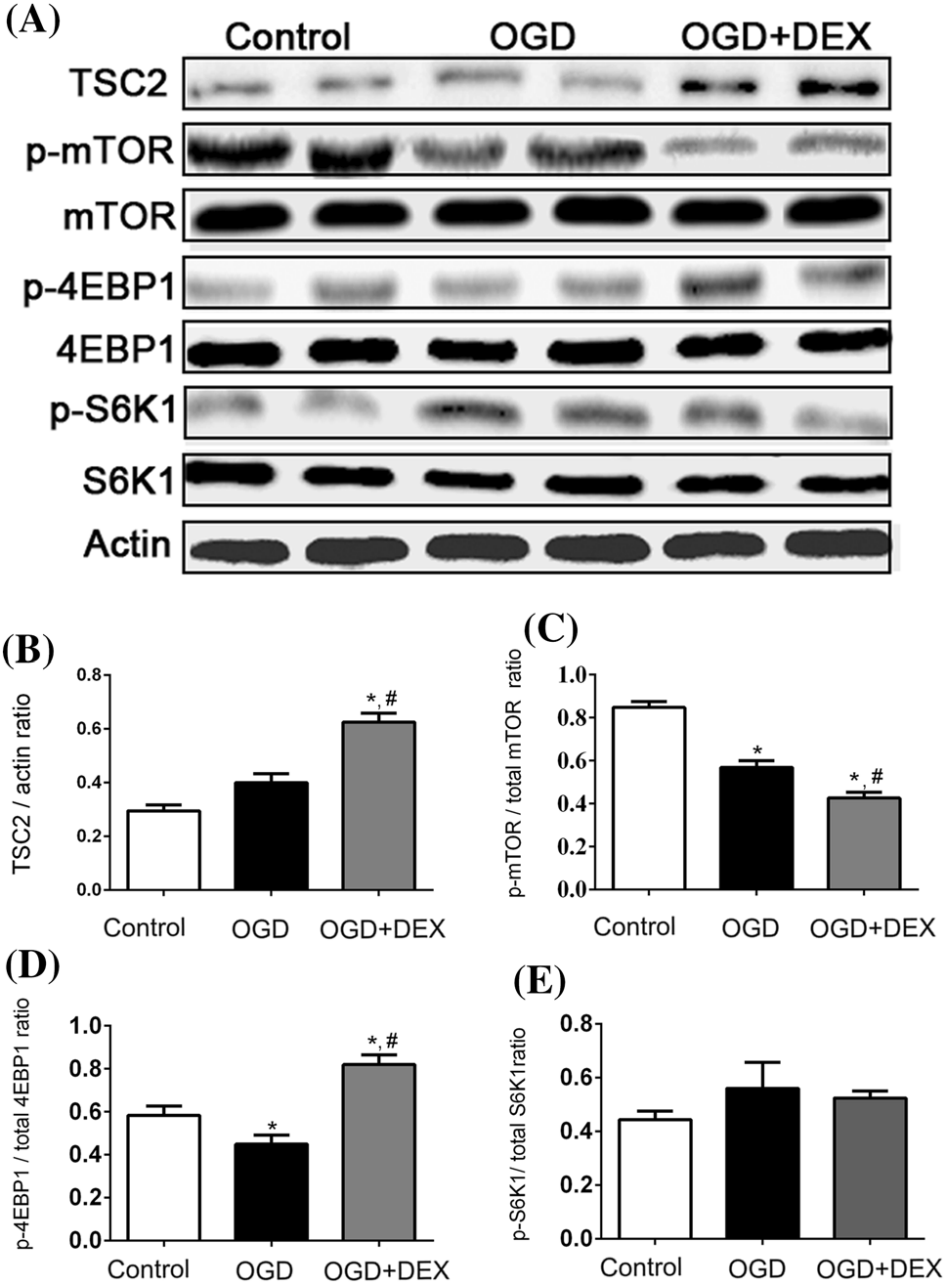
Dexmedetomidine induces astrocytes autophagy after OGD through TSC2/mTOR signaling pathway. **a** Representative immunoblotting image of TSC2, p-mTOR, mTOR, p-4EBP1, 4EBP1, p-S6K1, and S6K1 expression when treated with dexmedetomidine (DEX) following OGD. **b**, **c** Dexmedetomidine treatment upregulated TSC2 expression while downregulated p-mTOR expression following OGD. **d**, **e** Dexmedetomidine treatment upregulated p-4EBP1 expression, while not influencing p-S6K1 expression following OGD. *Compared with Control group, *P* < 0.05; ^#^Compared with OGD group, *P* < 0.05

To investigate which signaling pathway may relate with DEX-induced autophagy in astrocytes following OGD injury, we measured the expression level of TSC2, p-mTOR, mTOR, p-S6K1, S6K1, p-4EBP1, and 4EBP1 in different groups (Fig. 4a). The optical density of these proteins was normalized to that of the corresponding proteins in control group.

Our immunoblotting results suggested that DEX significantly elevated TSC2 expression in astrocytes exposed to OGD (Fig. 4b). Meanwhile, the phosphorylation of mTOR and 4EBP1 were markedly reduced and increased, respectively (Fig. 4c, d). However, DEX did not show an evident effect on phosphorylation of S6K1 (Fig. 4e).

## Discussion

**Fig. 5:**
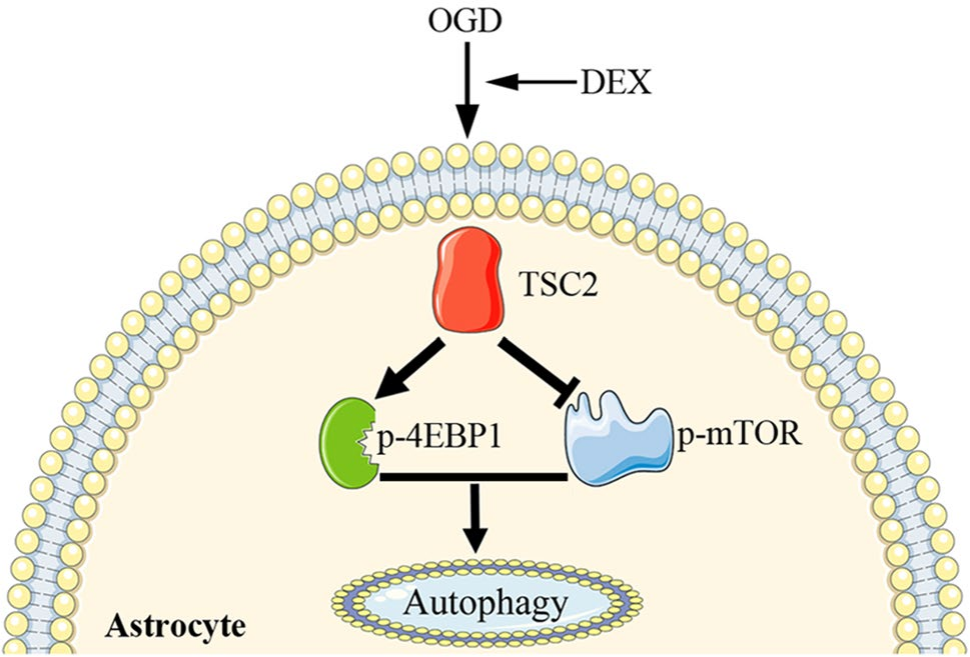
Schematic mechanism of DEX in regulation of astrocytes’ autophagy. DEX enhances astrocytes autophagy via upregulating TSC2/mTOR pathway

In the present study, we found that dexmedetomidine (DEX) could increase viability and inhibit apoptosis of astrocytes exposed to OGD. Our results also found that the protective effect may be related with DEX which further promotes the autophagy of astrocytes experiencing OGD. Furthermore, our results also found that DEX upregulated TSC2 expression and then decreased the phosphorylation mTOR. Additionally, the phosphorylation of 4EBP1 was also increased when treated by DEX. Thus, our in vitro study demonstrated that DEX promotes autophagy to protect astrocytes exposed to OGD, and this protection may relate with the TSC2-mTOR-4EBP1 signaling pathway (Fig. 5).

Autophagy was activated in cerebral ischemia; however, whether it is beneficial or detrimental to the ischemic brain is unclear (Shintani and Klionsky 2004; Rami and Kogel 2008). We previously demonstrated that DEX protects against focal cerebral ischemia via inhibiting neurons autophagy (Luo et al. 2017). However, the potential mechanism of DEX on astrocytes after cerebral ischemia is still unclear. Qin et al. found that the ratio of LC3-II/LC3-I was significantly upregulated and peaked at 3 h after OGD. Meanwhile, a significantly increased number of autophagosomes was observed in astrocytes 3 h following OGD (Qin et al. 2010). Our results also showed that the protection of DEX on astrocytes viability significantly improved at the 3 h reperfusion injury after OGD. And the protective effect may be related with DEX-induced autophagy of astrocytes. Many other researchers also found that autophagy may contribute to the neuroprotection in cerebral ischemia (Carloni et al. 2010; Wang et al. 2012, 2014). On the contrary, Qin et al. found that inhibition of astrocytes autophagy can mildly increase cell survival following ischemia–reperfusion injury (Qin et al. 2010). Therefore, autophagy may play different roles in different neural cells (e.g. neurons, astrocytes, microglia) when experienced with cerebral ischemia and subsequent reperfusion. Zhang et al. found that primary cultured neurons were exposed to 3-MA during the ischemia–reperfusion after 2 h or 4 h OGD showing decreased or increased cell viability, respectively (Zhang et al. 2013). Thus, the detailed effect and mechanism of autophagy in different timepoints during ischemia–reperfusion injury still needs further investigation.

The idea that DEX provides its neuroprotection via α_2_-adrenoceptors is occupying a central role to prevent ischemia–reperfusion injury is now generally accepted (Ma et al. 2004; Gu et al. 2011). However, DEX protection was also blocked by the I1-imidazoline receptors or I2-imidazoline receptors. (Ma et al. 2004; Dahmani et al. 2008). Our recent study also found that DEX protects against focal cerebral ischemia via promoting the HIF-1α expression in neurons (Luo et al. 2017). Otherwise, DEX can increase the expression of phosphorylated extracellular signal-regulated kinase 1/2 (ERK1/2) or active PI3K/AKT pathway or inactive TLR-4 signaling pathway in cerebral ischemia/reperfusion injury, which suggests that many other pathways may be related to DEX-induced neuroprotection (Ma et al. 2004; Dahmani et al. 2008; Kim et al. 2017). In present study, we investigated the relationship between DEX’s protection on astrocytes and the TSC2/mTOR signaling pathway, which is closely associated with autophagy. Mammalian target of rapamycin (mTOR), which negatively regulate autophagy, plays a vital role in cerebral ischemic reperfusion injury and is now generally accepted (Gabryel et al. 2012; Hayakawa et al. 2016). Our results found that TSC2, which can regulate the phosphorylation of mTOR, was upregulated by DEX and p-mTOR exhibited a corresponding decreased expression pattern. Furthermore, the phosphorylation of downstream proteins, such as 4EBP1 and S6K1, also showed a corresponding increased or decreased expression pattern respectively. Our results are in accordance with studies indicating that TSC2 inhibits phosphorylation of mTOR, and then leads to 4EBP1 activation (Roux et al. 2004; Carriere et al. 2011).

We previously demonstrated that DEX protects against focal cerebral ischemia via inhibiting neurons autophagy by promoting the HIF-1α expression (Luo et al. 2017). In present study, we found that the DEX could promote or enhance autophagy in astrocytes after OGD through TSC2/mTOR pathway, which could increase viability and inhibit apoptosis of astrocytes experienced 3 h after OGD. In our two studies, DEX plays a contrary effect on neuronal autophagy and astrocyte autophagy. Wu et al. demonstrated that RAPA could increase neurons viability, while 3-MA further aggravated impairments at reoxygenation 24 h after OGD treatment (Wu et al. 2018a). Another study showed that RAPA treatment reduced the infarct volume in 3d MCAO rats (Wu et al. 2018b). Therefore, this phenomenon may indicate that autophagy not only play different roles in the progress of cerebral ischemia but also exerts diverse effect in different cell types. Anderson et al. indicated that astrocytic scars formation supports growth of injured CNS axons via upregulating specific chondroitin sulfate proteoglycans (Anderson et al. 2016). Moreover, we found that in the middle cerebral artery occlusion model in mice, astrocyte-deficit mice were found with enlarged brain infarct size (Li et al. 2008). Reactive astrocytes contribute to direct reprogramming into neurons at the injury surrounding sites after brain injury or change own molecular expression and morphology from all forms of CNS insults, which are beneficial or detrimental to the surrounding neural and non-neural cells (Sofroniew 2009; Robel et al. 2011). Therefore, there are still many unravel functions of astrocytes in cerebral ischemia that needs further investigation. Together, our results suggest that DEX neuroprotection may also relate with enhanced astrocytes autophagy via TSC2/mTOR pathway.

In conclusion, we found that DEX could enhance autophagy to increase astrocytes survival. And the underlying mechanism may relate with DEX regulating TSC2/mTOR pathway. Our results indicate a different effect of DEX on neurons and astrocytes. In vivo or in clinical settings, astrocytes respond profoundly to neuronal injury or survive during the brain ischemic process. Hence, our future experiments will attempt to clarify the relationship between enhancing autophagy in astrocytes and inhibiting autophagy in neurons when treated by DEX in cerebral ischemic reperfusion injury.
